# Non-chronic kidney disease-induced calciphylaxis: a rare case report

**DOI:** 10.1093/jscr/rjae404

**Published:** 2024-06-04

**Authors:** Drake Giese, Katherine Ernste, Geethu Jnaneswaran, Ali Elkhedr

**Affiliations:** Medical College of Wisconsin – Central Wisconsin, Department of Education, 1900 Westwood Dr Suite 3100, Wausau, WI 54401, United States; Medical College of Wisconsin – Central Wisconsin, Department of Education, 1900 Westwood Dr Suite 3100, Wausau, WI 54401, United States; Marshfield Clinic, Division of Education - 1R61000 North Oak Avenue Marshfield, WI 54449, United States; Marshfield Clinic, Division of Education - 1R61000 North Oak Avenue Marshfield, WI 54449, United States

**Keywords:** calciphylaxis, calcific uremic arteriolopathy, non-chronic kidney disease, skin ulcers

## Abstract

Calciphylaxis is a disorder causing ischemic skin necrosis, typically associated with end-stage renal disease or those receiving dialysis. Occurrence is rare in those without end-stage renal disease, and treatment options are limited. This case report describes a patient with calciphylaxis without end-stage renal disease or history of dialysis. Treatment with sodium thiosulfate, a first line option, had to be stopped due to metabolic derangements, limiting the healing process. Diagnosis and treatment of this rare disorder are important to prevent further complications that may result.

## Introduction

Calciphylaxis is a rare and severe disorder causing ischemic skin necrosis, characterized by histological identification of dermal vessels and subcutaneous tissue calcification leading to ulceration and necrosis accompanied by intimal hypertrophy and small vessel thrombosis [1, 2]. It is most common in patients with end-stage renal disease (ESRD), or those receiving dialysis, but also possible in kidney transplant patients or in rare cases, those without ESRD. Calciphylaxis in those without ESRD is a poorly understood disorder with little information on potential etiologies.

## Case report

Here, we describe a case of a 65-year-old female with a history of calciphylaxis and associated chronic nonhealing wound of the right lower extremity, with intermittent infectious wounds and ensuing antibiotic treatment. She had previously been treated with sodium thiosulfate (STS) but was due to an acute kidney injury, hypokalemia, and metabolic acidosis.

**Figure 1:**
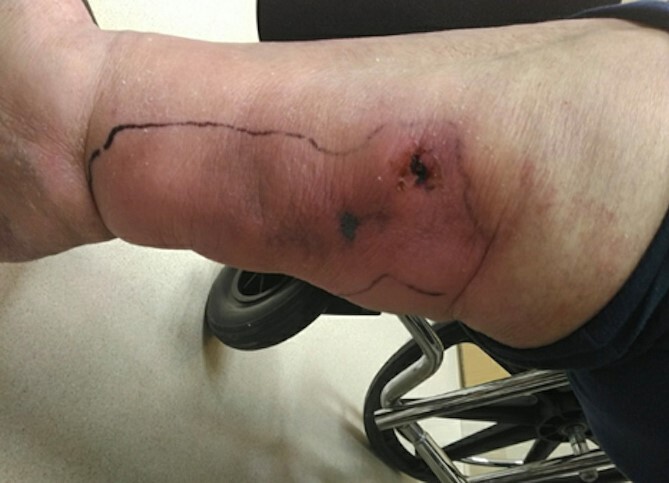
Initial presenting wound on medial aspect of right lower extremity.

After discontinuation, she presented to the hospital with progressively increasing pain in her wound with tracts extending superiorly from the wound. On presentation, patient had leukocytosis with predominant neutrophilia and elevated C-reactive protein and anemia secondary to chronic inflammation. All aerobic, anaerobic, and fungal cultures were negative. Patient and wound care team noted worsening since discontinuation of STS therapy with wound being more violaceous, non-viable appearing and of thrombosed nature. No abscess or fluid collection was found (Fig. 1).

**Figure 2:**
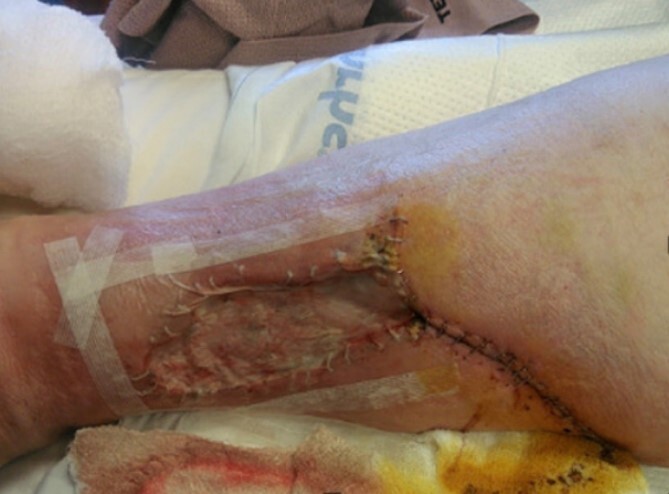
Wound post skin graft and debridement.

During hospitalization, the patient received excisional wound debridement of her right lower extremity and negative pressure wound therapy dressing was placed. Two days post debridement, a split thickness skin graft was performed (Fig. 2). Despite these efforts, the patient’s wounds grew. Due to negative cultures, worsening calciphylaxis was suspected versus infection. Dermatology advised restarting STS infusions, given patient’s interval normalization of parameters, and wound progression. The patient and wound team noted marked improvement while on STS therapy. The patient stabilized and her creatinine, blood urea nitrogen, sodium, potassium, and carbon dioxide remained within normal limits. Patient was discharged home with a pain management regimen and follow-up appointments with dermatology and the wound care team.

**Figure 3:**
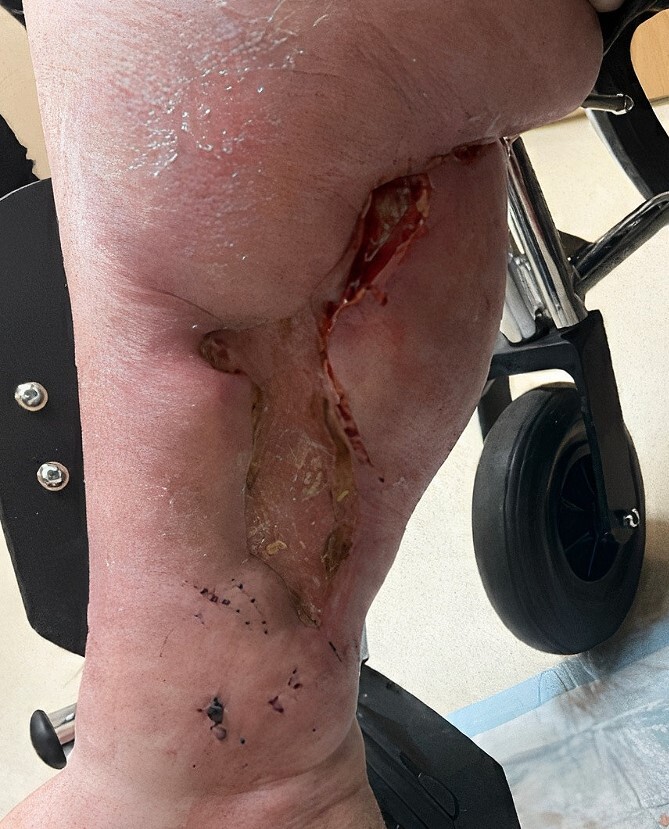
Three months postskin graph and reinitiating of STS therapy.

The patient initiated 12-week STS infusion cycle for her non-uremic calciphylaxis with close monitoring for any complications. She was found to have significant improvement of her inferior wounds, with re-epithelialization in 3 months by the end of the infusion cycle (Fig. 3). Unfortunately, she did have deterioration of her other chronic conditions and died from cardiac arrest after completion of her treatment.

## Discussion

Nonuremic calciphylaxis (NUC) and uremic calciphylaxis present similarly, in that there is a characteristic painful skin lesion that starts as red and reticular, advancing to necrosis with black eschar and surrounding purpura [3]. Lesions heal poorly and have high risk of infection [2, 3]. The diagnosis is made via cutaneous biopsy, which shows calcification of the tunica media, fibrointimal hyperplasia of small dermal and subcutaneous arteries along with thromboses in the arterioles leading to cutaneous ischemia and panniculitis [2, 3]. To differentiate between NUC and uremic calciphylaxis, laboratory studies assessing renal function must be completed.

In the literature, there are limited cases of NUC, also known as non-ESRD calciphylaxis. NUC is associated with a high mortality rate of 52%, most often due to sepsis [3, 4]. Mineral abnormalities prior to onset of symptoms are often absent, which may suggest a heterogenous mechanism of pathophysiology; however, the current mechanism is uncertain [3, 5]. There are several factors associated with NUC, most notably diabetes mellitus, malignancy, osteoporosis, autoimmune and granulomatous diseases, and prothrombotic conditions [3]. In this case, risk factors for calciphylaxis included female gender, obesity, history of systemic lupus erythematous, and diabetes mellitus [3, 6].

Treatment is uncertain for NUC, but most strategies follow guidelines for uremic calciphylaxis. The first step is identifying any triggers of occlusion or factors involved in the mineralization process [3]. Surgical or chemical debridement of the lesion is recommended to remove the affected tissue, with appropriate analgesic control [3]. A novel treatment for uremic calciphylaxis is STS, but this has not been studied in NUC [3]. Other therapeutic strategies for uremic calciphylaxis include hyperbaric oxygen therapy, lanthanum carbonate, statins, bisphosphonates, cinacalcet, sevelamer, and tissue plasminogen activator therapy [3, 7].

In our patient, she had no history of ESRD on diagnosis; however, due to limited treatment options available, STS was initiated to try and stop the progression of the disease. This was initially discontinued due to acute kidney injury, hypokalemia, and metabolic acidosis. However, with reinitiating treatment and close monitoring, the patient successfully had epithelialization of the affected area after 3 months. This suggests a potential role for STS in the treatment of NUC, although evidence remains limited.

This case report highlights a rare occurrence of non-CKD induced calciphylaxis in a patient with multiple comorbidities. Although calciphylaxis is primarily associated with end-stage renal disease, healthcare providers should consider this diagnosis in patients without CKD who present with painful skin ulcers and necrosis. Prompt recognition and management, including wound care and treatment of underlying conditions, are crucial for improving outcomes in these patients. Further research is needed to better understand the pathophysiology and risk factors associated with non-CKD–induced calciphylaxis; however, the success of STS in this patient may suggest potential treatment options for NUC cases in the future.
